# Osteoporosis and fracture after gastrectomy for stomach cancer: A nationwide claims study

**DOI:** 10.1097/MD.0000000000010532

**Published:** 2018-04-27

**Authors:** Gi Hyeon Seo, Hae Yeon Kang, Eun Kyung Choe

**Affiliations:** a Health Insurance Review and Assessment Service; b Department of Internal Medicine, Healthcare Research Institute, Seoul National University Hospital Healthcare System Gangnam Center; c Department of Surgery, Healthcare Research Institute, Seoul National University Hospital Healthcare System Gangnam Center, Seoul, South Korea.

**Keywords:** fracture, gastrectomy, osteoporosis, stomach cancer

## Abstract

This study was planned to evaluate the incidence and risk factors of osteoporosis and fracture after gastrectomy for stomach cancer using a nationwide claims database in South Korea.

Data from 41,512 patients (50–79 years) who underwent gastrectomy for stomach cancer from 2008 to 2010 with at least 5 years of follow-up were obtained from the Health Insurance Review and Assessment Service database. Patients diagnosed with osteoporosis and prescribed bisphosphonate or raloxifene or who experienced osteoporotic fractures after gastrectomy were operationally defined as osteoporosis. Osteoporotic fracture was defined as a fracture at common osteoporotic fracture sites (spine, pelvis, hip, forearm, or rib).

In total, 37,076 patients were included in the final analysis. The incidences of postgastrectomy osteoporosis and osteoporotic fractures were 41.9 and 27.6 cases per 1000 person-years, respectively. Multivariate analysis showed that older age (hazard ratio [HR] 1.88; 95% confidence interval [CI] 1.79–1.96), female gender (HR 2.46; 2.35–2.58), total gastrectomy (HR 1.10; 1.04–1.16), and diabetes (HR 1.16; 1.11–1.22) were significantly associated with osteoporosis and that older age (HR 1.90; 95% CI 1.80–2.01), female gender (HR 1.50; 1.41–1.58), total gastrectomy (HR 1.17; 1.10–1.25), chemotherapy (HR 1.06; 1.00–1.12), and diabetes (HR 1.26; 1.19–1.33) were significantly associated with fractures. Osteoporotic fractures occurred a median 3.1 years after gastrectomy. Among the 5175 fracture patients, 780 (15.1%) experienced multisite fractures, mostly in the elderly and chemotherapy groups.

The osteoporosis and osteoporotic fracture incidences are high in patients within a relatively short timeframe after gastrectomy for stomach cancer. Systematic management of osteoporosis is necessary after this surgery.

## 1 Introduction

Globally, stomach cancer is the fourth most common cause of cancer,^[1,2]^ and the early detection and postoperative survival rates are also increasing.^[3]^ In Korea, approximately 20,000 people undergo gastrectomy for gastric cancer every year. Osteoporosis and fractures commonly occur after gastrectomy for stomach cancer.^[4–6]^ In previous studies, the incidence of osteoporosis was reportedly 32% to 42%, and the incidence of fracture was approximately 40% after gastrectomy.^[4,6,7]^ Osteoporotic fractures interfere with patients’ quality of life and increase the socioeconomic burden on individuals and society.^[8]^

Despite the high prevalence of osteoporosis, no program has been established after gastrectomy for stomach cancer. In addition, the precise incidence is unknown, because many people are diagnosed with osteoporosis and fractures at hospitals outside of the hospital that performed the gastrectomy. The purpose of this study was to evaluate the incidence and risk factors of osteoporosis and fracture after gastrectomy for stomach cancer using the nationwide claims database in South Korea.

## 2 Patients and methods

### 2.1 Data acquisition

All South Koreans are obliged to enroll in the National Health Insurance Corporation; thus, approximately 98% of the Korean people are registered. Claims data from the Health Insurance Review and Assessment Service (HIRA) are collected when medical providers provide services to patients and request reimbursement from the HIRA. The patient records in the HIRA database include gender, age, diagnoses, treatments, and prescriptions.^[9]^ The data used in this study were obtained from all claims data registered between January 2007 and December 2015. Data prior to 2007 were inaccessible.

### 2.2 Study population

**Figure 1:**
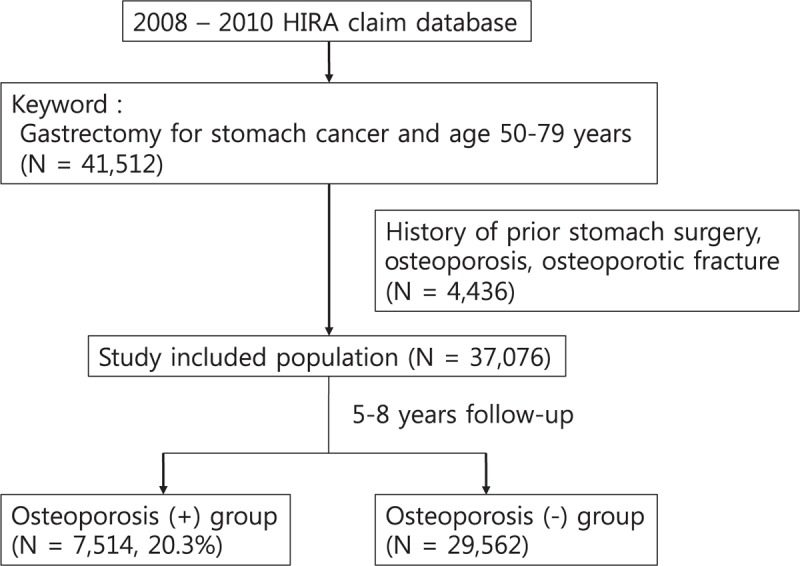
A flow chart of the study population. Patients were classified into the osteoporosis (+) group or fracture (+) group if they were diagnosed with operationally defined osteoporosis or fracture during the follow-up period after the index gastrectomy; otherwise, the patients were assigned to the osteoporosis (−) group or fracture (−) group.

Patients who underwent gastrectomy and had a diagnosis of stomach cancer (C160–C169 according to the ICD-10 classification) between January 2008 and December 2010 were identified from the HIRA database. These 54,146 patients were defined as having undergone index gastrectomy. Among these patients, individuals 50 to 79 years of age were selected for the analysis (n = 41,512). The average age of menopausal women in Korea is 50 years old; therefore, this study included only postmenopausal women 50 years of age or older. We excluded patients with a record of stomach surgery (n = 9), patients diagnosed with osteoporosis and prescribed bisphosphonates or raloxifene (n = 2458), or patients diagnosed with osteoporotic fractures (n = 2484) prior to the index gastrectomy. Finally, 37,076 patients were included in the final analysis (Fig. 1).

Because the HIRA data did not include bone mineral density (BMD) measurements using dual energy X-ray absorptiometry (DXA), an accurate osteoporosis diagnosis could not be obtained using T-scores.^[10,11]^ In Korea, there is a risk of overestimation when the diagnosis is considered, because the diagnostic code of osteoporosis can be used even in the diagnostic work-up process to apply for medical insurance. To avoid overestimation, we operationally defined osteoporosis as a diagnostic code for osteoporosis (M80–M82 according to the ICD-10 classification) plus a prescription for bisphosphonate or raloxifene or the occurrence of osteoporotic fractures after index gastrectomy. We included only bisphosphonates and raloxifene as the drugs for osteoporosis, because these drugs are most commonly used to treat osteoporosis in Korea. Moreover, hormone therapy or calcium agents could be prescribed for other indications. Osteoporotic fracture was defined as a fracture of a common osteoporotic fracture site, such as the spine, pelvis, hip, forearm, or rib.

We included the patient age at the time of the index gastrectomy (i.e., 50–64 years was considered young, and 65–79 years was considered elderly), patient gender, surgical method (subtotal gastrectomy [STG] vs. total gastrectomy [TG]), chemotherapy use (within 180 days after index gastrectomy or until osteoporosis or a fracture occurred), and diabetes mellitus status. Each patient underwent a follow-up period of at least 5 to 8 years, because we used data registered in the HIRA database until December 2015. The end of follow-up date was defined as the last claims data registered in the HIRA database, because most cancer patients were monitored for recurrence at least once yearly after the index gastrectomy for as long as the patients survived. Person-years were counted from the date of gastrectomy until the end of follow-up.

### 2.3 Ethics statement

The study protocol was approved by the Institutional Review Board of Seoul National University Hospital (IRB number 1706-054-858), and the study was conducted in agreement with the Declaration of Helsinki. Informed consent was waived by the board.

### 2.4 Statistical analysis

We expressed categorical variables as frequencies with percentages. The chi-squared test was used to assess differences between independent groups. The annual cumulative incidence rate for osteoporosis or fracture among the at-risk patients was calculated using Kaplan–Meier plots. Cox proportional hazard analyses were also performed to identify the risk factors for osteoporosis or fracture development. The statistical analyses were performed using the R statistical software, version 3.2.2 (R development Core Team; R Foundation for Statistical Computing, Vienna, Austria). Statistical significance was established for 2-sided *P* values < .05.

## 3 Results

### 3.1 Baseline characteristics

A total of 37,076 patients (9915 females, 26.7%) were analyzed in this study. The mean patient age was 63.4 ± 7.8 years, and 38.2% had diabetes. STG was performed in 76.7% of the patients, and 40.3% patients received chemotherapy. The patients were followed for 179,421 person-years. Our study had a mean follow-up period of 5.0 ± 2.3 years (median 5.7 years). Of these patients, 3690 (10.0%) were prescribed bisphosphonates or raloxifene, and 5175 patients (14.0%; 12.3% of the males and 18.4% of the females) had identified osteoporotic fractures after gastrectomy during the follow-up period (mean ± standard deviation: 3.3 ± 2.0 years, median 3.1 years, interquartile range 1.6–4.8). A total of 7514 (20.3%) of the patients were operationally diagnosed with osteoporosis after the index gastrectomy in this study.

### 3.2 Cumulative incidences of osteoporosis or fractures during the follow-up after the index gastrectomy

The 5-year cumulative incidence rates of osteoporosis for older age, female gender, TG, chemotherapy, and diabetes were 25.0% (vs. 13.7%, younger age), 31.2% (vs. 14.0%, male), 19.3% (vs. 18.6%, STG), 18.3% (vs. 18.9%, without chemotherapy), and 20.9% (vs. 17.4%, nondiabetes), respectively (Table 1). The multivariate cox regression analysis showed that older age (hazard ratio [HR] 1.88; 95% confidence interval [CI] 1.79–1.96), female gender (HR 2.46; 2.35–2.58), TG (HR 1.10; 1.04–1.16), and diabetes (HR 1.16; 1.11–1.22) were significantly associated with osteoporosis. Chemotherapy was not significantly related to osteoporosis.

**Table 1:**
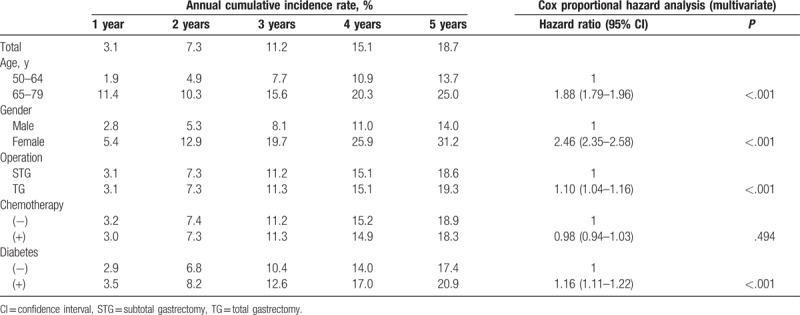
Annual cumulative osteoporosis incidence after gastrectomy calculated from Kaplan–Meier plots and cox proportional hazard analysis for each risk factor.

The 5-year cumulative incidence rates of osteoporotic fractures for older age, female gender, TG, chemotherapy, and diabetes were 17.1% (vs. 9.1%, younger age), 16.3% (vs. 11.3%, male), 14.0% (vs. 12.3%, STG), 13.1% (vs. 12.4%, without chemotherapy), and 14.8% (vs. 11.4%, nondiabetes), respectively (Table 2). The multivariate cox regression analysis showed that older age (HR 1.90; 95% CI 1.80–2.01), female gender (HR 1.50; 1.41–1.58), TG (HR 1.17; 1.10–1.25), chemotherapy (HR 1.06; 1.00–1.12), and diabetes (HR 1.26; 1.19–1.33) were significantly associated with fractures.

**Table 2:**
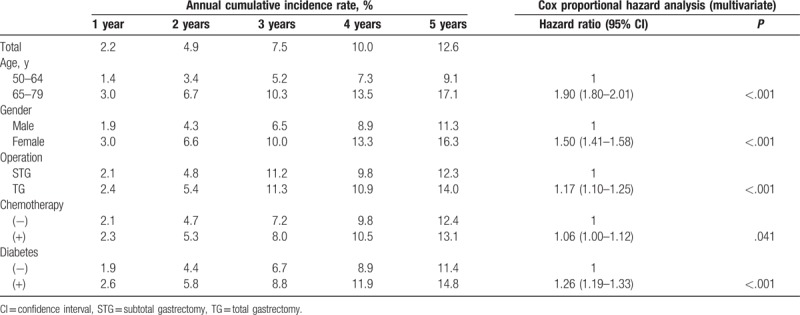
Annual cumulative osteoporotic fracture incidence after gastrectomy calculated from Kaplan–Meier plots and cox proportional hazard analysis for each risk factor.

### 3.3 Locations of fractures and multisite fractures after gastrectomy

Osteoporotic fractures were common in the spine (2031, 5.3%), rib (1966, 5.3%), forearm (1318, 3.6%), and femur (753, 2.0%). When we divided the patients by gender, rib fractures were the most common sites in males (5.5%), and spine fractures were the most common sites in females (13.1%). Among the total of 5175 fracture patients, 780 (15.1%) experienced multisite fractures (Table 3). Significantly more multisite fractures occurred in the elderly and chemotherapy groups (*P* < .05).

**Table 3:**
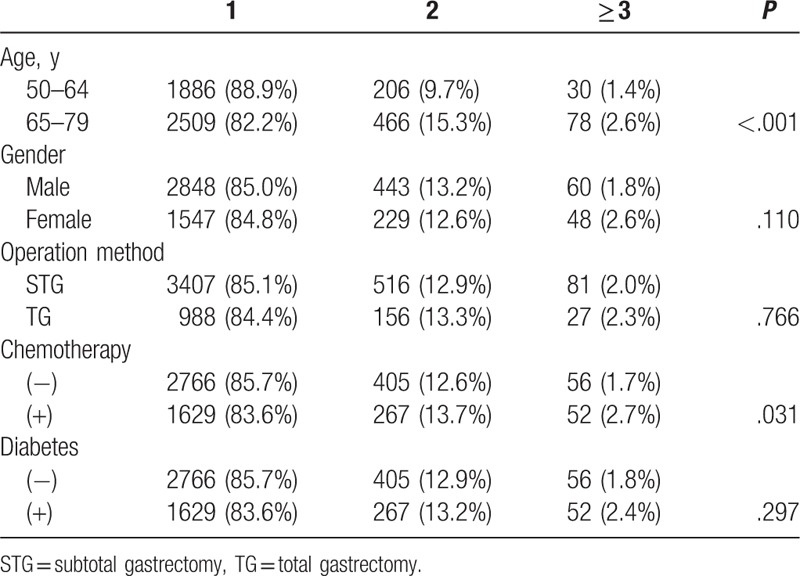
Multisite fracture (%).

## 4 Discussion and conclusion

Stomach cancer is particularly common in East Asia.^[12]^ Recently, a large proportion of stomach cancer cases have been diagnosed and treated early; as a result, the 5-year survival rate for early gastric cancer in Korea is over 90%.^[13,14]^ Therefore, more attention should be directed toward preventing osteoporosis to improve patients’ quality of life and reduce socioeconomic costs during the long survival period after gastrectomy. To the best of our knowledge, this study is the first to use nationwide claims data to investigate the incidence and risk factors of osteoporosis and fractures in patients who have undergone gastrectomy for gastric cancer. Older age, female gender, TG, and diabetes were significantly associated with osteoporosis and fractures. The elderly and chemotherapy groups were significantly associated with multisite fractures.

Although the pathogenesis is still unclear, malabsorption is known to be a common cause of osteoporosis after gastrectomy.^[15,16]^ Gastrointestinal physiology could be altered after gastrectomy and reconstruction. The primary sites affected after gastrectomy are the duodenum and proximal jejunum, which are the main sites of calcium absorption.^[4,17]^ Previous experimental studies have suggested that hypovitaminosis D and a subsequent increase in parathyroid hormone may contribute to increased rates of bone loss after gastrectomy.^[18–20]^ After gastrectomy, the patients experienced rapid weight loss of 5% to 15% during the immediate postoperative period, which also affected the risk of osteoporosis and fractures due to a lack of appetite, dyspepsia, and altered intestinal motility.^[21,22]^

In a previous study, the prevalence of osteoporosis among patients 50 years of age or older was 32.9% to 35.5% for females and 7.5% to 12.2% for males according to the BMD, and the treatment rate was 44% to 58% for physician-diagnosed osteoporosis patients in Korea.^[23–25]^ A previous study also reported a higher incidence of osteoporosis, because the authors examined every patient who underwent gastrectomy for osteoporosis regardless of the symptoms.^[4]^ Osteoporosis diagnoses were made using the World Health Organization (WHO) T-score of <−2.5, which was calculated from the Asian reference data. However, prior to October 2011, the South Korean reimbursement guidelines for osteoporosis treatment required that patients to have a T-score of <−3.0 or a history of osteoporosis-related fractures of the spine or hip. Thus, based on the WHO and reimbursement guidelines, some real patients may not have been included in our study. Bisphosphonates and raloxifene are the most commonly used drugs to treat osteoporosis, and their market share among nonhormonal osteoporotic drugs reaches approximately 90% in Korea.^[26,27]^ Therefore, osteoporosis might be underestimated in this study, because many people were not diagnosed due to the lack of symptoms. In addition, many diagnosed patients were not treated, and people who used drugs other than bisphosphonate or raloxifene were excluded. However, various factors, such as the definition of osteoporosis, the methods used to determine the BMD, and the reference population used to calculate the T-scores, affect the incidence rate of osteoporosis.^[24,28,29]^ In this study, we used an operational definition of a patient with newly diagnosed osteoporosis (with a prescription) after index gastrectomy.

In the present study, osteoporotic fractures were identified in 12.3% of males and 18.4% of females after index gastrectomy. Female gender is a risk factor for both osteoporosis and fracture. Fracture may also be underestimated, because fractures are included only in cases diagnosed in a hospital. In fact, the fracture incidence rates were relatively low compared to the rates found in a previous single-center study that reported a cumulative incidence of fracture up to 40.6%, with most fractures occurring within the first 6 postoperative years.^[6]^ In that study, annual bone scintigraphy with additional computed tomography, magnetic resonance imaging, or X-ray examinations were performed with or without trauma; thus, those results differ from our research findings. In our study, most patients might have clinically important symptoms or trauma to receive medical treatment. Moreover, fractures were identified within a median of 3.1 years after the index gastrectomy in this study because a bone remodeling imbalance reportedly occurs early within the first postoperative year.^[30]^

In this study, diabetes, TG, and chemotherapy were associated with a higher risk of fracture. Type 2 diabetes is a well-known cause of secondary osteoporosis.^[31]^ Patients who underwent TG were more likely to have more advanced disease, receive more chemotherapy, and consequently have more severe weight loss than patients who underwent STG. Chemotherapy can also be related to osteoporotic fractures because it induces physical inactivity and weight loss due to the lack of appetite and reduced oral intake and the effects of the chemotherapeutic drugs themselves. Systemic chemotherapeutic agents, such as 5-fluorouracil and cisplatin, which are the most commonly used drugs for gastric cancer, have been reported to contribute to bone loss by inducing apoptosis of osteoblasts and increasing osteoclast activity.^[32–34]^

The American Gastroenterological Association has recommended DXA in patients who are at least 10 years postgastrectomy, particularly postmenopausal females and males over 50 years of age, based on reports involving patients with peptic ulcer disease.^[35–38]^ However, the application of this recommendation to patients with gastric cancer is inappropriate because cancer patients tend to be older and have worse general conditions than peptic ulcer patients. Moreover, the risk of osteoporosis or fracture in the short term after surgery should be considered, as described our study. A program designed to diagnose and prevent osteoporosis and fractures should be established shortly after surgery for gastric cancer patients who are expected to survive long term.

This study has several limitations. First, an accurate osteoporosis diagnosis using T-scores was not possible, because the HIRA data did not include the BMD. Thus, the incidence of osteoporosis reported in this study might differ from the incidence reported in studies that defined osteoporosis based on BMD scores. Second, the HIRA database does not include laboratory data, BMI, weight change, menopause, smoking, or the cancer stage, which are known risk factors for osteoporosis. The average age of menopause in Korean women is 50 years old; therefore, this study included only patients older than 50 years of age at the time of the index gastrectomy. Third, fractures in this study might include bone metastasis or trauma, other than osteoporotic fracture, even if we only included common osteoporotic fracture sites. Fourth, the duration after gastrectomy is not sufficient, because the HIRA database has been available only since 2007. Therefore, our study had a mean follow-up period of only 5.0 years. Nevertheless, our results confirmed that the fracture incidence rate was high during the initial follow-up years.

In conclusion, this study demonstrated a high prevalence of osteoporosis and fractures in stomach cancer patients in the early postgastrectomy years. A careful surveillance program is needed early after gastrectomy to prevent and detect osteoporosis early and to prevent fractures for the improvement of the long-term quality of life of gastric cancer survivors. Previous studies have suggested that bisphosphonate therapy shows effectiveness in increasing the BMD and reducing the risk of fracture in gastric cancer patients after gastrectomy.^[39,40]^ Further studies are needed to compare the incidence of fractures between osteoporosis patients treated with bisphosphonate or other active agents and those who are not treated. A systematic program should be developed to prevent and manage osteoporosis and fractures through periodic follow-up after gastrectomy for stomach cancer.

## Author contributions

**Conceptualization:** Gi Hyeon Seo, Hae Yeon Kang, Eun Kyung Choe.

**Data curation:** Gi Hyeon Seo, Hae Yeon Kang, Eun Kyung Choe.

**Formal analysis:** Gi Hyeon Seo, Hae Yeon Kang.

**Investigation:** Gi Hyeon Seo, Hae Yeon Kang.

**Methodology:** Gi Hyeon Seo, Hae Yeon Kang.

**Project administration:** Hae Yeon Kang.

**Resources:** Hae Yeon Kang.

**Software:** Gi Hyeon Seo, Hae Yeon Kang.

**Supervision:** Hae Yeon Kang, Eun Kyung Choe.

**Validation:** Hae Yeon Kang, Eun Kyung Choe.

**Visualization:** Hae Yeon Kang, Eun Kyung Choe.

**Writing-original draft:** Hae Yeon Kang.

**Writing-review and editing:** Gi Hyeon Seo, Hae Yeon Kang, Eun Kyung Choe.
